# Mechanism of Genome Editing Tools and Their Application on Genetic Inheritance Disorders

**DOI:** 10.1055/s-0044-1790558

**Published:** 2024-09-16

**Authors:** Dae Hwan Oh

**Affiliations:** 1 Institute of Green Manufacturing Technology, Korea University, Seoul, Republic of Korea

**Keywords:** gene therapy, hemophilia, cystic fibrosis, Duchenne muscular dystrophy, CRISPR, base editor, prime editor

## Abstract

In the fields of medicine and bioscience, gene editing is increasingly recognized as a promising therapeutic approach for treating pathogenic variants in humans and other living organisms. With advancements in technology and knowledge, it is now understood that most genetic defects are caused by single-base pair variants. The ability to substitute genes using genome editing tools enables scientists and doctors to cure genetic diseases and disorders. Starting with CRISPR (clustered regularly interspaced short palindromic repeats)/Cas, the technology has evolved to become more efficient and safer, leading to the development of base and prime editors. Furthermore, various approaches are used to treat genetic disorders such as hemophilia, cystic fibrosis, and Duchenne muscular dystrophy. As previously mentioned, most genetic defects leading to specific diseases are caused by single-base pair variants, which can occur at many locations in corresponding gene, potentially causing the same disease. This means that, even when using the same genome editing tool, results in terms of editing efficiency or treatment effectiveness may differ. Therefore, different approaches may need to be applied to different types of diseases. Prevalently, due to the safety of adeno-associated virus (AAV) vectors in gene therapy, most clinical trials of gene therapy are based on AAV delivery methods. However, despite their safety and nonintegration into the host genome, their limitations, such as confined capacity, dosage-dependent viral toxicity, and immunogenicity, necessitate the development of new approaches to enhance treatment effects. This review provides the structure and function of each CRISPR-based gene editing tool and focuses on introducing new approaches in gene therapy associated with improving treatment efficiency.

## Introduction

CRISPR, or clustered regularly interspaced short palindromic repeats, is a mechanism of the prokaryotic adaptive immune system. The application of CRISPR-associated proteins (Cas) along with recombinant DNA technology has revolutionized the fields of medicine and molecular biology by transforming it into a powerful gene editing tool. Over time, CRISPR/Cas technology has rapidly evolved, leading to the emergence of new versions of genome editing tools. Notably, base editors (BEs) and prime editors (PEs) are in the spotlight for their ability to edit genomes without creating double-strand breaks (DSBs), making them potent tools for gene therapy. 1 2 Due to their remarkable ability to specifically edit genes in organisms such as humans, mammals, and plants, CRISPR-based genome editing tools have become a promising approach for treating genetic disorders, including hemophilia, cystic fibrosis, and Duchenne muscular dystrophy (DMD), which are characterized by variants in specific gene regions and have hereditary traits.

Regarding hereditary diseases, more than 7,000 rare diseases are recognized globally, and according to the National Organization for Rare Disorders, hundreds of millions of patients suffer from these conditions, with two-thirds being children. 3 While a few drug-based treatments like antisense oligonucleotides (ASOs) are approved to alleviate symptoms, no existing treatments or therapies address the underlying genetic variants for more than 95% of these patients. Even with available drug-based treatments, patients must continue medication for life, as these do not correct the fundamental genetic issues.

However, the emergence of CRISPR-related gene therapy has made it possible to correct these fundamental genetic variants and has been applied for permanent gene modification. Furthermore, based on the drawbacks of the necessity for iterative medication and the display of a range of symptom severities, hemophilia, cystic fibrosis, and DMD have become prime candidates for CRISPR-related gene therapy. The severity of hemophilia ranges from mild to severe, depending on the clotting factor level caused by various types of variants, such as gene deletions, missense variants, and nonsense variants. Moreover, the severity of cystic fibrosis is categorized into six classes, which are also caused by similar types of variants as those found in hemophilia. Lastly, a broad range of gene deletions causes DMD, resulting in progressive muscle loss or mild symptom called Becker muscular dystrophy (BMD). Not only are these three disorders hereditary, but their wide range and various types of variants also make them suitable candidates for gene correction using CRISPR-related therapy. Therefore, by correcting the underlying variants that cause these diseases, there will be no possibility of passing down the diseases to future generations and no necessity to devise treatments for each variant. Owing to its safety features, such as nonintegration into the host genome and relatively low immunogenicity, most clinical trials for these genetic disorders have utilized gene therapy methods employing adeno-associated virus (AAV) as a delivery vector. Nevertheless, limitations such as dosage-dependent toxicity, potential for immune responses, low transduction efficiency due to preexisting neutralizing antibodies (Nabs), and limited loading capacity pose challenges. Scientists are actively seeking ways to overcome these obstacles to enhance the effectiveness of gene therapy for genetic diseases. 4 5 6

This review focuses on explaining the mechanisms of gene editing tools and their application across various approaches to overcome the limitations of AAV-mediated gene therapy, ultimately aiming for higher treatment efficiency. While it primarily addresses overcoming the limitations of using AAV-mediated gene therapy, it also introduces approaches that enhance the efficacy of these AAV-mediated strategies.

## Genome Editing Tools

### CRISPR/Cas System

**Fig. 1:**
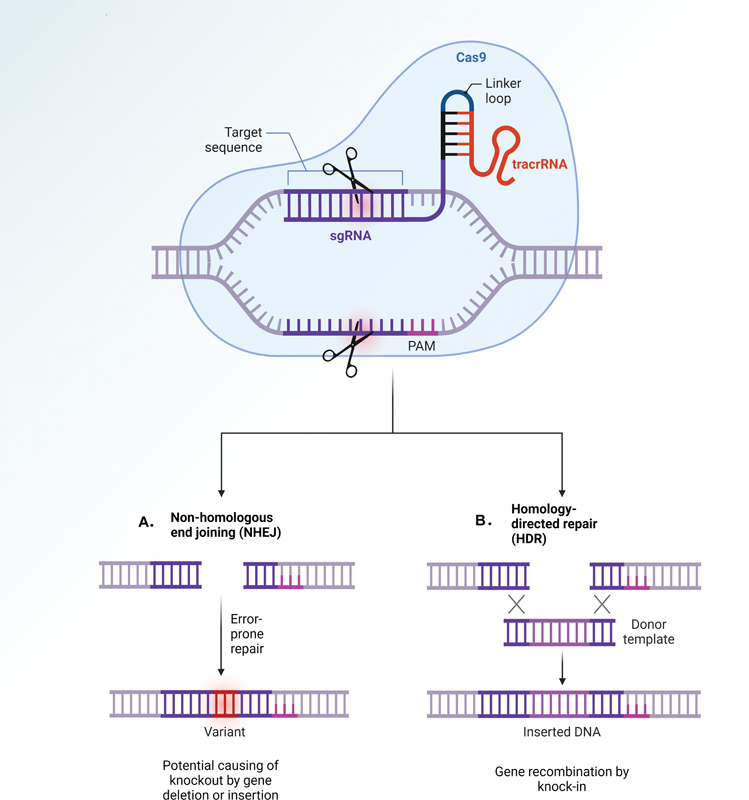
Mechanism of the CRISPR/Cas system. ( **A** ) Nonhomologous end joining (NHEJ): this pathway ligates the ends of broken DNA without a homologous template, potentially leading to nucleotide deletions or insertions. NHEJ can be activated in both dormant and proliferating cells. ( **B** ) homology-directed repair (HDR): HDR is a DNA repair process that repairs double-strand breaks (DSBs) through homologous recombination using a DNA template. The HDR pathway is primarily activated during the G2 and S phases of the cell cycle. The most common application of this pathway in biology, utilizing the CRISPR/Cas system, is to knock in a target gene into a specific sequence for recombination (created with BioRender.com).

The CRISPR/Cas system is derived from the immune defense mechanisms of bacteria and archaea, which fight against foreign genetic materials from various sources, including bacteriophages, transposons, and plasmids. 7 The system consists of sgRNA—a fusion of crRNA that binds to the target sequence and tracrRNA, which maintains the activation of the Cas protein—and the Cas protein itself, a nuclease that cleaves DNA and creates DSBs. 8 Before the creation of a DSB, the gRNA guides the Cas protein to a “protospacer” located near a suitable protospacer adjacent motif (PAM). 9 The introduction of the DSB by the Cas protein triggers the DNA repair system, either through nonhomologous end-joining (NHEJ) or homology-directed repair (HDR). In the case of NHEJ, the cleaved DNA ends rejoin directly, potentially causing knocked-out genes or frameshift variants due to random nucleotide insertions or deletions ( Fig. 1A ).

Conversely, if a homologous donor template is present, the DSB initiates a precise HDR pathway, resulting in a knock-in 10 11 ( Fig. 1B ). Furthermore, genome editing is conducted differently based on the type of target nucleic acid and is applied with different Cas protein complexes; the most utilized tools are Classes 1 and 2. While Class 1 is composed of multisubunit effector complexes containing multiple Cas proteins, Class 2 systems involve a single Cas protein and are commonly used in the fields of biological and medical research.

### Base Editing

**Fig. 2:**
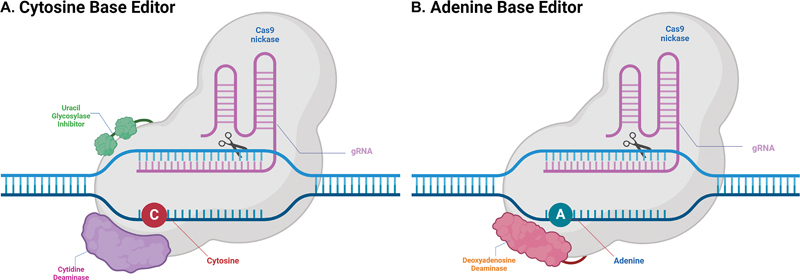
Components and mechanisms of CBE (cytosine base editor) and ABE (adenine base editor). ( **A** ) CBE: cytidine deaminase mediates the deamination of cytosine to uracil. An uracil glycosylase inhibitor (UGI) prevents the removal of uracil by uracil DNA glycosylase (UDG) during the base excision repair pathway. ( **B** ) ABE: deoxyadenosine deaminase mediates the deamination of adenosine, resulting in the generation of inosine, which is recognized as guanine by DNA polymerase (created with BioRender.com).

As aforementioned, the DNA repair system is activated, leading to two pathways: HDR and NHEJ. A major concern with this genome editing tool is the potential for causing undesired insertions and deletions (indels), which can deteriorate cellular conditions through mechanisms such as the activation of p53 and undesired translocations. Consequently, one alternative strategy to overcome the limitations of the CRISPR/Cas system is BE. 12 13 Instead of using Cas9, the BE technique employs Cas9 nickase, which cleaves only a single strand of DNA. 14 This technique also utilizes another protein enzyme that modifies a single nucleobase—cytidine deaminase and deoxyadenosine deaminase. 15 Cytidine deaminase is an enzyme that catalyzes the hydrolytic deamination of cytosine to uracil. To prevent the removal of the replaced uracil, cytidine deaminase includes an additional protein called uracil glycosylase inhibitor, which protects uracil from uracil-DNA glycosylase. As the Cas9 domain opens the target DNA site, creating a bubble, all cytosines in the unpaired DNA are exposed to cytidine deaminase, resulting in the deamination of cytosine within roughly a five-base window, creating a U:G mismatch. 16 To induce the cell to replace the G (guanine) on the nontargeted strand with A (adenine), the BE nicks the G-containing strand, leading the cell to use the uracil-containing strand as the template. If the conversion process proceeds correctly, the C–G base pair will be successfully replaced with a U–A or T–A base pair ( Fig. 2A ).

Similarly, adenine base editor (ABE) operates like cytosine base editor (CBE) but uses deoxyadenosine deaminase instead of cytidine deaminase. When applied to a targeted strand containing A (adenine), deoxyadenosine deaminase catalyzes the hydrolytic deamination of adenine to yield I (inosine), which is recognized as G (guanine) in DNA system. Using the same DNA repair method as CBE, the A:T base pair is converted to G:C ( Fig. 2B ).

However, even though the BE has a low risk of producing indels and demonstrates high performance in nucleotide substitution, it still poses a potential risk of undesired editing. This is caused by limitations in targeting due to confined PAM requirements, bystander editing related to the width of the activity window, and possible off-target effects. 17

Overall, BE avoids the risk of DSBs, and the editing efficiency of nucleotide substitution performed by BEs in plants and animals is significantly higher than that of HDR-mediated single base pair correction. 1

### Prime Editing

**Fig. 3:**
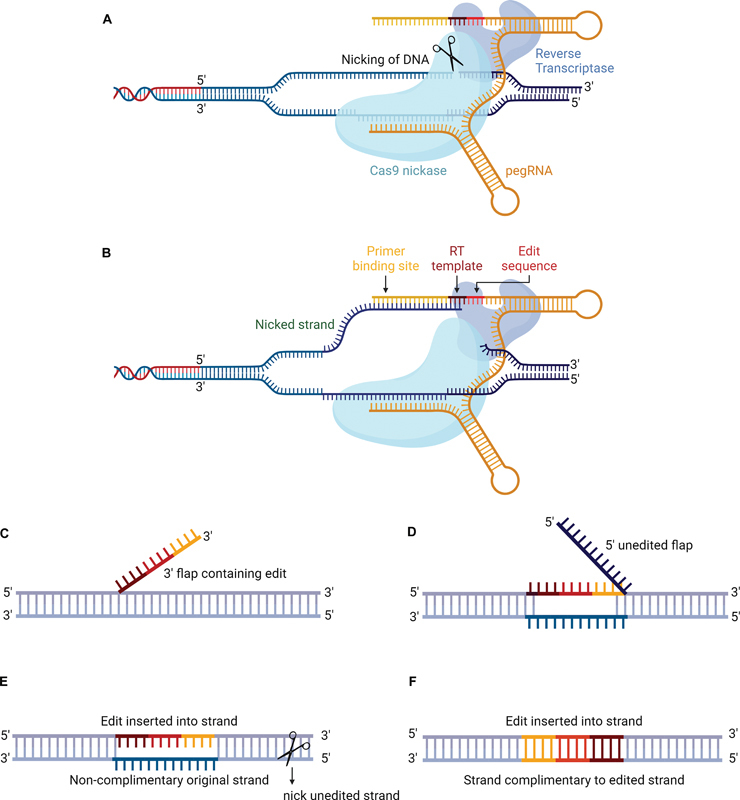
Mechanism of prime editor. ( **A** ) Components of prime editing and Cas9 nickase cleaves one strand of DNA. ( **B** ) PegRNA function: PegRNA binds to the primer binding site (PBS) of the nicked strand, and reverse transcriptase (RT) begins to synthesize the desired cDNA sequence based on the RT template. ( **C** , **D** ) Strand Integration: the synthesized 3′ flap containing the edited strand takes over after the 5′ flap containing the unedited strand is removed by 5′ exonucleases. ( **E** ) Additional Cleavage: cleavage of the nonedited complementary original strand triggers the substitution of the edited strand. ( **F** ) DNA repair mechanism: the desired edited strand is introduced by the DNA repair mechanism (created with BioRender.com).

One notable difference between the standard CRISPR/Cas system and PE is the presence of reverse transcriptase (RT), which is an essential component of the PE system. The PE consists of three parts: a fusion of Cas9 nickase and an engineered RT protein, a prime editing guide RNA (pegRNA), and a nicking guide RNA (ngRNA) ( Fig. 3A ). The pegRNA guides the fusion protein to bind with a target DNA strand, which is then nicked by the Cas9 nickase. Once hybridized with the nicked strand, the pegRNA serves as a template for reverse transcription, leading to the creation of a flap that plays a crucial role in precise genetic conversions ( Fig. 3B ). This involves accepting a newly created 3′ strand flap and degrading the original 5′ strand 18 19 ( Fig. 3C, D ).

To enhance the efficiency rate of genetic alteration, the ngRNA guides the fusion protein to the strand opposite the flap-containing strand and nicks the nonedited strand. This increases the probability of repairing the nonedited strand, ultimately resulting in a completely edited homoduplex double-stranded DNA 1 20 ( Fig. 3E, F ).

PE was developed to overcome the limitations of BEs and the CRISPR/Cas system. It can convert genomes with almost any intended changes, including all 12 types of transition and transversion point variants, as well as insertions and deletions (indels), without requiring DSBs or a donor DNA template. Furthermore, it is reported that PE has the potential to correct up to 89% of more than 75,000 disease-associated variants in humans. 21 However, despite its advantage of processing genome editing without a DSB or donor DNA, many challenges, such as determining the off-target level, have not yet been fully addressed. 2

## Genetic Disorders Targeted for Gene Therapy and Comparison between Editing Efficiency of Those Genome Editing Tools Used in Gene Correction

Many therapy attempts are focused on using the genome editing tools described above. Specifically, the most targeted and ongoing treatments for genetic disorders are for Hemophilia, cystic fibrosis, and DMD. Below, I will provide examples of treatments using gene editing methods and compare the efficiency of these treatments across different genome editing tools, including CRISPR/Cas, BEs, and PEs.

### Hemophilia

Hemophilia A (HA) is an X-linked recessive hereditary bleeding disorder caused by a deficiency in coagulation factor VIII (FVIII) due to a variant in the F8 gene, located at Xq28. 22 Hemophilia B (HB) is also an X-linked recessive hereditary bleeding disorder caused by a deficiency in coagulation factor IX, resulting from a variant in the F9 gene, located at Xq27. Furthermore, both HA and HB are caused by various variants affecting the entire regions of the FVIII and FIX genes. Specifically, inversion variants in intron 22 and intron 1 are responsible for 43% and 2% of severe HA cases, respectively. Additionally, HA is affected not only by inversions but also by a broad range of missense, nonsense, frameshift, indels, and splicing variants. On the other hand, inversion variants are not observed in HB; however, most patients exhibit mild or moderate symptoms resulting from missense variants. 23 The possibility of suffering from HA is approximately 1/5,000 and 1/25,000 in live male births. The severity of hemophilia differs by the plasma clotting factor levels. While patients with mild symptoms have >5% of normal clotting factor level, patients with residual factor level of >13% rarely go through severe joint bleeding. However, more than half of patients have blood factor level of <1% of normal. Before the gene therapy, while HA was mostly treated with replacement therapy using emicizumab, a bispecific monoclonal antibody that mimics the role of FVIII, 24 HB was generally treated with plasma-derived factor IX and recombinant factor IX. Furthermore, hemophilia has also been treated with antifibrinolytics, drugs like tranexamic acid or aminocaproic acid that are often used in conjunction with factor IX concentrates, and desmopressin, which is mainly used for mild HA and limited use in HB. 25 However, traditional treatments for hemophilia face limitations such as the short duration of therapeutic effects, frequent intravenous injections, short plasma half-life of recombinant coagulation factors, and the production of Nabs against these clotting factors, known as inhibitors. 26 Consequently, scientists and doctors have been searching for more effective and sustainable methods of treating hemophilia. 27 With the advent of CRISPR technology, the possibility of correcting genes has positioned hemophilia as a prime candidate for gene therapy. Commonly, due to its characteristics of existing as an episome after transduction, low immunogenicity, and efficient entry into quiescent cells, the AAV vector is employed in gene therapy to treat hemophilia. 28 Despite the many advantages of using AAV vectors, their limitations, including immunogenicity, preexisting Nabs, dose-dependent toxicity, and limited capacity, mean that gene therapy using AAV vectors is not yet perfectly administered. 29 30 31

#### CRISPR/Cas System

To increase the treating efficiency of hemophilia, unlike traditional gene therapies that target the F8 and F9 genes directly, 32 33 34 35 new approaches involve bypass gene correcting methods have been devised. Even though using AAV vector has some limitations as earlier mentioned, this bypass method of using AAV vector is used as permanent inserting gene into host cells, not as a common usage of episome, delivering the clotting factor mFVIIa to the Rosa26 locus, a safe harbor for expressing genes of interest in mice. 36 Sarangi et al used three AAV vectors: two AAV8 vectors to load gRNA and a donor template of mFVIIa, and one AAV2 to load SpCas9. 37 The editing efficiency showed frequencies of 20 to 42%, with a significant decrease in prothrombin time observed in mice with a 22% indel frequency, from 11.07s ± 0.86 to 8.62s ± 0.4. However, this approach is challenging to apply in clinical trials due to the incomparable level of FVIIa expression between patients with HA and mice, and there is a potential risk of thrombosis. 38

On the other hand, another bypass method uses the CRISPR/Cas system delivered by lipid nanoparticles (LNP) in vivo. Notably, instead of directly correcting the F8 or F9 genes, this method increases the activity of factor Xa and the generation of thrombin by creating frameshifts in the antithrombin (mAT) gene to suppress its protein expression levels. LNPs consist of ionizable cationic lipids, polyethylene glycol lipids, zwitterionic phospholipids, and cholesterol, enhancing the efficiency of genome editing by improving biocompatibility with anionic cell membranes and promoting endosomal membrane destabilization. 39 This approach has positive effects such as rapid cargo delivery, low immunogenicity, dose-dependent toxicity, and sophisticated target accuracy. Han et al injected LNP-CRISPR-mAT into mice with HA, caused by inversion in 22 intron of FVIII gene, and B, creating frameshift in SerpinC1 gene, encoding antithrombin III, resulting in indel frequencies of 22 and 38% in the liver tissues, respectively. Although this method successfully suppressed AT protein expression using innovative bypass strategies, it showed insufficient efficacy against external wounds due to the absence of clotting factors. 40

#### Base Editor

As for BE methods, an approach to treat HB used a CBE. In experiments using TAID-SpCas9-NG 41 with gRNA3, where TAID is a deaminase called PmCDA1, 42 showed notable results. After applying TAID-SpCas0-NG to HEK293 cells with an I316T variant in the F9 gene, FIX activity increased to 16.1 ± 2.3%, an average across eight clone cells with notably high measures. Further in vivo investigations involved injecting knock-in mice with human F9 carrying the I316T variant using two AAV vectors carrying TAID-SpCas9 with gRNA3 via an intein-mediated split method. 43 Consequently, FIX activity levels and FIX antigen expression increased to 3.8 and 2.4%, respectively, with an editing efficiency of 1.74 ± 0.31%. Even though this approach uses AAV vector, it solves the vector capacity problem by using intein-mediated split. 44

Meanwhile, another approach using the BE ABE8e and a nonviral vector was performed successfully by Rong et al. HEK293 cells and hepatocellular carcinoma cell line Huh7 with the R226Q point variant in exon 6 of the F9 gene, which causes abnormal FIX activity, 45 were observed to recover their FIX activity after transfection with PLL-plasmid nanoparticles carrying ABE8e. 46 The recovered FIX activity rates in HEK293 cells and Huh7 cells were approximately 38 and 49% of the wild type, respectively, 47 which are exceptionally high considering the normal FIX activity rate ranges from 50 to 150%. 48 This result could be significant for treating hemophilia if the safety conditions are fully determined.

Unfortunately, despite numerous examples of using CRISPR-related gene editing methods, including BEs, there has been no detectable approach that utilizes PEs for treating hemophilia.

### Cystic Fibrosis

Cystic fibrosis is an autosomal recessive genetic disease caused by variants in the CFTR (cystic fibrosis transmembrane conductance regulator) protein. CFTR is an ion channel protein responsible for channeling cyclic adenosine monophosphate (cAMP)-activated chloride (Cl ^−^ ) and bicarbonate (HCO3 ^−^ ) across epithelial membranes, thus modulating the flow of fluids such as mucus, sweat, saliva, tears, and digestive enzymes. Therefore, abnormalities in ion transport can lead to difficulty in water absorption, resulting in dehydration of the airway surface liquid, altered mucus composition, and compromised protection against bacterial infections. For example, increased viscosity of the mucus layer can lead to small airway obstructions and adhere to the apical membrane of epithelial cells, which could facilitate pathogen colonization. Eventually, a patient with cystic fibrosis could end up with severe respiratory failure, such as extensive bronchiectasis. 49 50 Cystic fibrosis is categorized into six classes based on symptoms, functions, and protein output. In normal individuals, CFTR protein is formed and transferred to the cell surface, where it controls the movement of chloride. People with cystic fibrosis class 1, accounting for 22% of patients worldwide, are unable to produce CFTR. Class 2, the most common among those with cystic fibrosis, accounting for 88%, can produce CFTR protein, but the protein is misfolded, which disrupts its transfer to the cell surface, known as a trafficking defect. Class 3, accounting for 6%, can produce and transfer the protein to the surface but has defective channel regulation, meaning the channel gate does not operate properly. Class 4, also accounting for 6%, operates similarly to Class 3, but with reduced functionality, resulting in defective chloride transport through the channel. Class 5, accounting for 5%, has normal CFTR protein but insufficient quantities. Class 6, also accounting for 5%, has normal ability to produce and transport the protein to the cell surface, but as the protein reaches the surface, its stability decreases, resulting in a reduced amount of protein at the cell surface. 51 As the symptoms differ, it is clear that targeting genomic sites and applying techniques vary as well. Specifically, diseases related to the airway epithelial cell type are very difficult to cure compared with other cell types affected by cystic fibrosis. Prevalently, AAV vector is considered to be the most suitable gene therapy to treat cystic fibrosis because of its efficiency of iterative dosing and safety. 52 53 However, due to its limitations, such as the low expression level of CFTR 54 55 and a limited carrying capacity of approximately 4.6 kb, many other gene therapy methods have been developed to treat cystic fibrosis.

#### CRISPR/Cas System

One treatment approach using CRISPR/Cas targets the 3849 + 10 kb C > T variant, which is a Class 5 cystic fibrosis variant that creates an undesirable TAA stop codon in intron 22 between exons 22 and 23. This approach uses a nanocomplex as a delivery to delete the stop codon. Since the airway epithelial cell is a dormant cell, its treatment is more adequately addressed using the NHEJ pathway rather than HDR, which is highly dependent on the cell cycle. The treatment approach involves eliminating 178 bp of intron 22, after which DNA repair occurs via the NHEJ pathway. To trigger the DNA repair pathway, ribonucleoprotein (RNP) complexes (Cas9 and two gRNAs) are delivered to cystic fibrosis basal epithelial cells by receptor-targeted nanocomplexes. These nanocomplexes consist of bifunctional peptides, featuring a cationic binding domain and cyclic peptide motif for cell targeting, and lipids comprising a one-to-one molar mixture of cationic DOTMA (1,2-dioleoyl-sn-glycerol-3-phosphoethanolamine) and DOPE (1,2-dioleoyl-sn-glycerol-3-phosphoethanolamine). This offers the advantage of low immunogenicity and facilitates both RNP packaging and receptor-mediated uptake. After four rounds of transfection, a 62% indel frequency is observed in alleles. This result demonstrates the potential of this approach, surpassing the recommended therapeutic levels of CFTR editing of higher than 25%. 56 Although this approach shows a relatively high efficiency rate of genome editing, it has not yet shown clinical usefulness in therapy. However, it is notable that using the air–liquid interface (API), which resembles human airway epithelial cells exposed to air on the apical side and to fluid on the basal side, donor-free repair and nonviral vectors seem to make genome editing relatively easier than other approaches. 57

#### Base Editor

An approach using BE ABE7.10, which converts adenine to guanine (A > G), is applied to correct the stop codon (TGA) of R553X variant causing class 1 cystic fibrosis, a severe disease that results in undetectable levels of CFTR mRNA and the absence of CFTR protein. 58 59 In this approach, two types of cell lines are used: the CuFi-3 cell line, an airway cell line that is compound heterozygous for ΔF508 and R553X variants typically used for cystic fibrosis-related experiments, 60 and primary human airway epithelial cells bearing R553X/G85E variants, both cultivated in an API to mimic the condition of airway epithelial cells. To increase editing efficiency and overcome the limitations of Cas protein recognizing the PAM sequence, a modified SpCas9 protein that recognizes the “NG” PAM sequence is used. 61 After the electroporation method, ABE7.10–SpCas9–NG is transmitted to both cells and shows an editing efficiency frequency of 82.1% in CuFi-3 cells and 54.5% in primary cells. Furthermore, in edited cells, activation and inhibition of CFTR are observed by adding forskolin and IBMX, and GlyH-101, respectively. 62 This approach utilized electroporation and RNP delivery methods, which avoid the immune responses associated with viral vectors and risks of prolonged or off-target effects. 63 Additionally, using a BE to correct genes in quiescent cells like airway epithelial cells demonstrates higher efficiency in genome editing and is more suitable than using the CRISPR/Cas system with the HDR pathway. 64

#### Prime Editor

As for using PE attempt to correct the CFTR W1282X variant involves. The W1282X variant is also a Class 1 cystic fibrosis variant that prevents the creation of any CFTR. To fix the W1282X variant, codon 1282, which codes for the amino acid tryptophan, needs to be changed from TGA to TGG, as TGA is a stop codon. However, it is noteworthy that due to the PE's large size, two to three lentiviruses or AAV are needed to deliver the PE machinery into cells. To overcome this limitation, the invention of helper-dependent adenovirus (HDAd), which has a large capacity of 37 kb and shows efficient transduction of airway cells and low toxicity by removing all viral coding sequences, has made it possible to deliver the PE machinery into host cells efficiently. 65 66 All pegRNA with an 11-nt primer binding site (PBS) and a 15-nt RT template, PE, and the nick gRNA(-37)-1 are packaged in an HDAd and then applied to induced pluripotent stem cell (iPSC)-derived airway epithelial cells. The result demonstrates that 71.7 ± 5.4% of cells received the PE machinery, but only approximately 2.4 ± 0.6% of cells were corrected. Although editing efficiency showed low-frequency results, in the Ussing chamber assay, the edited cells successfully showed an increase in Isc (short-circuit current) after stimulation with forskolin (FSK), which can raise the level of cAMP resulting in activation of CFTR, aligning with the expected CFTR function compared with the unedited iPSC-derived airway epithelial cell showing no response. Even though the PE is a safer genome editing tool that doesn't require any donor template or creation of DSBs, it is not yet permitted for clinical trials because of its significantly low overall editing efficiency. Since PE is a current genome editing technique, it seems to require more experiments and information before it is applied to clinical trials. 67 However, this approach showed the promising solution for capacity problem that delivery vector, including AAV vector, normally has.

### Duchenne Muscular Dystrophy

DMD is a progressive muscle degenerative disease that causes difficulties with movement, requires ventilation support, and ultimately leads to premature death due to heart failure. Predominantly affecting males, patients with DMD often become wheelchair-bound before the age of 12 and may pass away in their early 20s. This condition is an X-linked recessive disorder caused by variants in the *DMD* gene, responsible for encoding dystrophin, a protein vital for muscle function. These variants disrupt the production of the muscle isoform of dystrophin. Dystrophin is a rod-shaped cytoplasmic protein that links the cytoskeleton of a muscle fiber to the surrounding extracellular matrix through the cell membrane. This linkage is facilitated by the dystrophin-associated protein complex (DAPC). Without a properly formed DAPC, muscle tissue loses its functionality, leading to cardiomyopathy. 68

From a genetic standpoint, the *DMD* gene spans a full length of 11.4 kb and hosts four internal promoters—Dp260, Dp140, Dp116, and Dp71—that produce N-terminal truncated nonmuscle isoforms of dystrophin. Additionally, the Dp40 promoter initiates alternative splicing at the 3′-end and alternative polyadenylation, creating an additional isoform. The main domain of the *DMD* gene is segmented into four parts: the “N-terminal F-actin-binding domain” encoded by exons 1 to 8 (ABD), the “central rod domain” encoded by exons 8 to 64, the “cysteine-rich domain” encoded by exons 64 to 70 (CR), and the “C-terminal domain” encoded by exons 71 to 79 (CT). Moreover, the rod domain is composed of 24 spectrin-like repeats and four interspersed hinges. DMD manifests from frameshift variants that cause deletions or duplications in specific parts of the gene, and nonsense variants, which result in incomplete dystrophin proteins. Consequently, patients with DMD exhibit truncated exons, leading to a deficiency of dystrophin and a disruption in the connections between the cytoskeleton and the extracellular matrix. However, BMD, characterized by the presence of partially functional dystrophin that includes the main domains—ABD, CR, and CT—presents with milder symptoms of DMD. 69

As mentioned about BMD, it is notable to see that if *DMD* gene is truncated but maintaining the reading frame, dystrophin protein can still be partially functional resulting in mild symptoms of DMD. 70 71 72 By using this knowledge, methods of mitigating symptoms of DMD have been invented.

In recent developments, ASOs have emerged as a promising therapeutic option to address or alleviate the symptoms of DMD variants. ASOs, utilizing an exon skipping method, can restore a reading frame by skipping one or more exons, thereby producing functional dystrophin. Innovations in this area have led to the emergence of a new generation of ASO drugs, including peptide-conjugated phosphorodiamidate morpholino oligomers (P-PMO) and Tricyclo-DNA (tcDNA). 73 74

Although ASOs are considered one of the most promising methods for treating DMD, they require repeated administration as they do not permanently alter the underlying genetic defect. Consequently, permanent gene correction therapy is necessary for DMD patients. Commonly, the AAV vector is used as a delivery method for treating DMD due to its safety and stability. While there is currently a micro-dystrophin gene therapy that employs an AAV vector in an episomal form, 75 the approaches described here focus on gene correction methods, including exon deletion, reframing, and skipping.

#### CRISPR/Cas System

An experiment using the CRISPR/Cas system with an AAV delivery vector has been devised to correct the mutated *DMD* gene in an mdx mouse. This approach employs exon deletion to restore the reading frame of the *DMD* gene by deleting an exon where a nonsense variant has occurred. Two AAV8 vectors were employed: one vector contained two gRNAs targeting introns 22 and 23, respectively, and the other vector carried SaCas9, aiming to delete the mutated exon 23 using the NHEJ pathway. The editing efficiency achieved was approximately 2%, leading to restored dystrophin expression. This restoration significantly improved muscle morphology and enhanced both skeletal and cardiac muscle functions, with restored dystrophin protein at 8 and 67% of muscle fibers testing positive for dystrophin compared with the wild type. 76 This approach is recognized as one of the common gene therapy methods for treating DMD through exon deletion. To overcome the limited capacity of AAV vectors, Nelson et al utilized two AAV8 vectors. Although this strategy may address the capacity issue of AAV vectors, it introduces the potential for dosage problems, which could lead to viral toxicity and potentially result in low-editing efficiency.

On the other hand, the purpose of exon reframing and skipping is to restore a functional reading frame and produce a truncated yet functional dystrophin protein by creating indels in specific parts of the mutated *DMD* gene. To demonstrate this approach for clinical approval, an experiment was performed on a humanized DMD mouse model. This involved inserting human exon 51, commonly mutated in human DMD, and deleting mouse exon 50, by creating an indel with the NHEJ pathway between the premature termination codon 5′-TGA-3′, the PAM sequence for Cas9 protein in this experiment, and the 5′-AG-3′ splice acceptor. The result was a reframing by either one nucleotide insertion (3n + 1) or two nucleotides deletion (3n − 2). Additionally, the indel was large enough to potentially disrupt the 5′-AG-3′ splice donor, which could lead to the skipping of exon 51. To facilitate this, SpCas9-VRQR and two copies of sgRNA-9, recognizing the 5′-TGA-3′ PAM sequence in human exon 51, were incorporated into an AAV9 vector and a scAAV vector, respectively. These were then introduced into the tibialis anterior, triceps, diaphragm, and heart of the mDmd ΔEx50; hDMD Ex51 knock-in humanized DMD mouse model. As a result, dystrophin protein expression reached 18 to 25% of wild-type levels in all targeted tissues, with indel frequencies of 11, 15, 13, and 17% in the tibialis anterior, triceps, diaphragm, and heart, respectively. Reframing events—either one nucleotide insertion (+T) or two nucleotides deletion (−GT)—accounted for an average of 55 to 65%, and exon skipping events occurred at an average rate of 4.5 to 12.8%.

While this approach yielded notable results and addressed the issue of limited vector capacity, it cannot be universally applied due to potential safety challenges in clinical settings. The experiment indicated that alterations in amino acid residues around the reframed exon could generate neoantigens. Additionally, there is a risk of liver damage in large animals due to the injection of high doses of AAV, the viral vector used. 77 78 79

#### Base Editor

In their efforts to develop new treatments for DMD, Chemello and colleagues turned to advanced gene editing tools. They applied an ABE to a mouse model with exon 51 deletion in the *DMD* gene causing DMD (ΔEx51), which results in a stop codon in exon 52. Seeking to enhance the functionality of the dystrophin protein, albeit in a truncated form, they experimented with skipping either exon 50 or exon 52 by employing a single-swap base pair transition at either the splice donor site (SDS) or splice acceptor site of the introns.

After numerous trials to find the most effective gRNA and BE combination for this base transition, the researchers chose ABEmax–SpCas9–NG 41 80 and a sgRNA targeting the antisense strand of the SDS region of exon 50 (5′-AC-3′). This setup, which showed the highest editing efficiency among the various combinations tested, was then packaged into an AAV9 vector. Due to the size constraints of the AAV vector, which has a packaging limit of less than 5 kb and the ABEmax–SpCas9–NG BE size of 5.8 kb, a dual AAV9 system utilizing a split-intein system was used for delivery. 43 Each half of the ABEmax–SpCas9–NG was fused with halves of the DnaE intein from Nostoc punctiforme (Npu) 81 and then transduced into ΔEx51 mice.

The on-target editing efficiency in these experiments was approximately 35%, resulting in 54% of dystrophin expression relative to the wild type and restored 96.5% of myofibers. To see if this method could be applied to humans, 51-deleted human iPSC-derived cardiomyocytes (ΔEx51 iPSC) were generated. Using the same BE strategy of skipping exon 50, sgRNA-1 targeting the SDS of human DMD exon 50 and ABEmax–SpCas9 were injected into ΔEx51 iPSC, which showed a relatively high on-target efficiency of 87.7%. 82 The benefit of using SpCas9–NG is its more relaxed PAM recognition, which increases target availability. Furthermore, this approach does not cause DSBs and addresses the problem of vector capacity using a split-intein system. However, since the approach utilizes dual AAV vectors, it exceeds the Food and Drug Administration-recommended dosage for AAV vectors, which could lead to significant viral toxicity. 83

#### Prime Editor

Building on this, Chemello and the team explored using PE hEx52-PE and sgRNA-4, previously used in BE for its efficiency, to reframe exon 52 by inserting dinucleotides 5′-AC-3′ into its antisense strand. They also employed two types of sgRNA to increase editing efficiency: one causing a nick 29 nucleotides upstream (nick-1) and another 52 nucleotides downstream (nick-2) on the sense strand of the same cells. 2 They detected a 20.2% efficiency for introducing a +2-nt GT insertion using hEx52-PE with nick-1, and a 54.0% efficiency with hEx52-PE and nick-2. Compared with healthy control iPSC-derived cardiomyocytes, the expression of dystrophin protein was 24.8% with nick-1 and 39.7% with nick-2. Moreover, the percentage of arrhythmic calcium traces in these human cardiac iPSC models of DMD, which was approximately 64%, decreased to 38 and 41% after injection with hEx52-PE and nick-1, and hEx52-PE and nick-2, respectively. 82

Despite these promising results, the progression toward clinical trials has been complicated by uncertainties regarding the appropriate viral dosage, highlighting the need for more precise dosing strategies to ensure safety and efficacy in clinical applications.

## Discussion

CRISPR-related treatments represent a versatile and promising technology capable of addressing virtually any disease by correcting the genetic abnormalities that underlie specific conditions. As technology advances, we anticipate the development of more advanced genome editing tools, akin to the emergence of BE and PE. This review examines the mechanisms of these genome editing tools and their application in treating hereditary and typically genetic disorders.

In the context of hemophilia, there is currently no substantial evidence or experimental data to demonstrate effective treatment using PE. Instead, various alternative treatments have been devised, including the bypass method, which does not target the mutant gene directly. Similarly, for conditions such as cystic fibrosis and DMD, the bypass method employing ASOs has been used. 74 84 Nevertheless, this review emphasizes that most genome correction strategies for these diseases, including PE, focus on directly rectifying the responsible genes.

Furthermore, the methods described for enhancing treatment efficacy primarily aim to address the existing limitations of genome editing, such as immunogenicity, dosage-dependent toxicity of viral vectors, and limited vector capacity. For example, using a nanocomplex as a delivery vector could potentially mitigate the immunogenicity associated with viral vectors. However, while promising safety results in gene therapy have been demonstrated, challenges persist, particularly in using nanoparticles for treating respiratory diseases like cystic fibrosis. 85 Regarding PE, despite its potential to correct 89% of human genetic diseases due to its extensive editing range, the mechanisms and challenges impacting editing efficiency—including the length of the PBS and pegRNA, target cell type, and challenges in finding a suitable vector due to its full size—remain inadequately understood. 86

It's important to note that overcoming one limitation does not always guarantee desired outcomes. Numerous factors must be considered, including the choice of vector (e.g., lentivirus, AAV, or nonviral vectors), chromatin accessibility, cell cycle presence, the length of the donor template, the type of Cas protein used, and cell types. This review does not account for all these variables or extensively compare results. It primarily focuses on enhancing treatment efficiency through methods that address the limitations of current gene therapy, predominantly using AAV vectors. Therefore, for a comprehensive comparison of editing efficiencies and to achieve high treatment outcomes as described here, one must refer to further studies and conduct additional investigations.

Moreover, for those researching ways to enhance editing efficiency, it is encouraging to note that many novel methods and technologies are continually being developed, potentially leading to significant therapeutic outcomes. 87 88 89 90 91
